# The PI3K/AKT Pathway and *PTEN* Gene Are Involved in “Tree-Top Disease” of *Lymantria dispar*

**DOI:** 10.3390/genes13020247

**Published:** 2022-01-27

**Authors:** Fengjiao Li, Long Liu, Xiao Yu, Christopher Rensing, Dun Wang

**Affiliations:** 1 State Key Laboratory of Crop Stress Biology for Arid Areas, College of Plant Protection, Northwest A&F University, Yangling 712100, China; fengjiao831@foxmail.com (F.L.); dragonliulong@foxmail.com (L.L.); yuxiaomichael@foxmail.com (X.Y.); 2 Institute of Environmental Microbiology, College of Resource and Environment, Fujian A&F University, Fuzhou 350002, China; crensing94@gmail.com

**Keywords:** tree-top disease, behavior change, PI3K/AKT signaling pathway, *Lymantria dispar*

## Abstract

Nucleopolyhedrovirus (NPV) can alter its host behaviour such that infected larvae hang at the top of trees before their death. This phenomenon was firstly described by Hofmann in 1891 and named as “tree-top disease”. Subsequent studies have described effects during the infection proceedings as NPVs manipulate the host to avoid the immune response, cross defensive barriers and regulate hormones. In this study, we demonstrate that the phosphatidylinositol 3-kinase (PI3K)/protein kinase B (AKT) pathway is involved in host manipulation by Lymantria dispar multiple nucleopolyhedrovirus (LdMNPV). Particularly at the late stage of infection, a multifunctional dephosphorylase in the PI3K/AKT signaling pathway is dynamically upregulated, namely, the phosphatidylinositol-3, 4, 5-trisphosphate 3-phosphatase and dual-specificity protein phosphatase (*PTEN*) gene. The biological assays of *PTEN* gene knockdown showed that an increase in *PTEN* gene expression was necessary for the infected *Lymantria dispar* larvae’s terminal climbing behavior, death postponement and virion production. The results imply that the PI3K/AKT signaling pathway and *PTEN* gene might play an essential role in “tree-top disease” induced by LdMNPV.

## 1. Introduction

Many parasites manipulate their hosts in various ways via changes to gene expression, cellular metabolism, signal transduction and even behavior to increase fecundity and transmission rates [1,2,3]. Baculoviruses provide an example of parasite-induced alteration of host behavior and are characterized by a strong behavioral change whereby the infected larvae usually climb to an elevated location before death [4,5,6]. The phenomenon that baculovirus-infected larvae climb to the top of trees before their death was first observed by Hofmann and was named “Wipfelkrankheit”: tree-top disease [5]. Nevertheless, not all insects alter their behavior by climbing to an elevated location after baculovirus infection [7,8]. The enhanced locomotory activity of silkworm after infection with Bombyx mori nucleopolyhedrovirus (BmNPV) occurs without the climbing phenomenon [8].

Baculoviruses are large DNA viruses with circular, double-stranded genomes, which specifically infect arthropods and have a unique mode of replication [6,7,9]. Behavioral changes of infected larvae may be accomplished by complicated interactions, such as changes in gene expression, altered hormone regulation and metabolism [10,11]. The unique replication model of baculoviruses and the individual physiological status of the insect causing the interaction are difficult to decipher. Existing studies merely suggested that the viral gene encoding protein tyrosine phosphatase (PTP) was required for inducing the host behavioral change in group I alphabaculoviruses, as in BmNPV and Autographa californica multiple nucleopolyhedrovirus (AcMNPV) [11,12]. In group II alphabaculoviruses, ecdysteroid uridine 5′-diphosphate (UDP)-glucosyltransferase (*egt*) encoded by the viral gene was involved in LdMNPV- and Spodoptera exigua multiple nucleopolyhedrovirus (SeMNPV)-triggered climbing behavior [13,14]. Moreover, baculoviruses developed photolyase-like genes, which have UV-induced DNA repair activity and circadian adjustment function [15]. Additional studies have shown changes in the insect larvae between the virus and host interaction. Comparing the differentially expressed genes (DEGs) of brains that were infected with BmNPV and healthy silkworms, it was shown that BmNPV-induced hyperactive behavior was correlated with synaptic transmissions, serotonin receptor signaling and circadian rhythm pathways [16]. It was reported that in LdMNPV-infected hyperactive larvae, the photosensitivity was increased and the rhythmicity-relevant genes, especially period (*per*) and timeless (*tim*), were impacted [17]. The process of the baculovirus manipulating the infected host to produce progeny is seemingly simple. However, intricate and complex fluctuations in the cell program and cellular signaling transduction have been demonstrated.

As a nonliving entity, the virus requires manipulation of the cellular program upon successful infection to replicate and survive. Studies have uncovered parts of the signaling manipulation of host-encoded genes by which baculoviruses progress during infection. Previous studies suggested that the viral fibroblast growth factor (vFGF) of baculoviruses stimulated tracheoblast growth in the secreting midgut epithelium to cross the barrier [18]. To avoid the DNA damage response of the host triggered by virus replication, baculoviruses contain genes encoding apoptosis suppressors to block apoptosis of the infected cell [19,20,21].

Insects are able to receive environmental stress and, after going through cellular signals, regulate physiological processes such as postembryonic development, reproduction and ecdysis. During the lifecycle, corresponding stimulation of the central nervous system (CNS) causes secretion of hormones, such as steroids, 20-hydroxyecdysone (20E) and juvenile hormone (JH), to activate the insulin-like signaling pathway and downstream phosphorylation signal, such as protein kinase B (AKT), Forkhead Box O (FoxO) and target of rapamycin (TOR), to trigger appropriate responses [22]. It has previously been shown that the insulin-like/phosphatidylinositol 3-kinase (PI3K)/AKT signaling pathway plays an important role in insect diapause, energy storage, cellular regulation and lifespan [23].

A number of pathogens, especially viruses, have been recognized to reprogram cellular processes by hijacking host cellular signals to create the required environment. As reported, the hepatitis C virus (HCV) activates the PI3K/AKT signaling pathway to assist viral replication and host cell survival [24]. As Mazzon et al. reported, Semliki Forest virus (SFV) infection increases glycolysis by hyperactivation mediated by the PI3K/AKT signaling pathway [25].

In the current study, it was uncovered that the PI3K/AKT pathway was involved in host manipulation by LdMNPV, and that the upregulation of the phosphatidylinositol-3, 4, 5-trisphosphate 3-phosphatase and dual-specificity protein phosphatase (*PTEN*) gene was necessary for virion production, host insect death postponement and the climbing behavior of terminally infected larvae.

## 2. Materials and Methods

### 2.1. Insect and Virus

*L. dispar* eggs were obtained from Professor Liang-Jian Qu from the Chinese Academy of Forestry, Beijing, China. Hatched larvae were reared on an artificial diet in a sterile phytotron under conditions of a photoperiod of 14:10 h (light/dark), temperature 26 ± 1 °C and relative humidity 60 ± 5%.

LdMNPV was stored in the laboratory. The occlusion bodies (OBs) were obtained from liquefied larvae and prepared using the following steps: The dead bodies were washed with 0.25% sodium dodecyl sulfate (SDS) and filtered through cheesecloth and filter paper. The filtrate was washed twice with double-distilled water, then centrifuged at 3000 rpm for 30 min and resuspended in double-distilled water. The resuspended precipitate was quantified using a bacterial counting chamber and then diluted to a final concentration of 10^6^ OBs/mL. The suspension was stored at 4 °C for further use.

### 2.2. Sample Preparation

Newly molting third-instar larvae were randomly divided into an infection group and a control group. The infection group was fed with LdMNPV OBs at a concentration of 10^6^ OBs/larva. The same volume of double-distilled water was fed to the control group at the same way. After feeding, larvae were reared in individual wells of a 24-well insect rearing box with ample artificial diet in each well. The larvae from each group were retained to confirm the treatment was working. Samples of the infected group were killed followed by liquefaction, and larvae of the control group were observed until pupation. The sampled larvae were snap frozen in liquid nitrogen and then stored at −80 °C for subsequent steps.

### 2.3. Quantitative Real-Time RT-PCR Analysis of Core Genes in the PI3K/AKT Pathway

Three larvae from the each group were collected separately every 8 h and were mixed together as one replicate. Each biological sample consisted of three replicates. Total RNA was extracted using TRIzol reagent (Invitrogen, Carlsbad, CA, USA) following the manufacturer’s protocols. The quality of RNA samples was determined using 1% agarose gel, and the concentration was measured using a NanoDrop 2000 spectrophotometer (Thermo Fisher Scientific Inc., Waltham, MA, USA). Total RNA was treated with DNase I (TaKaRa Bio Inc., Otsu, Shiga, Japan) to remove the residual genomic DNA. Each sample was reverse transcribed into cDNA using PrimeScriptTM RT Reagent Kit Perfect Real Time (TaKaRa, Bio Inc., Otsu, Shiga, Japan) according to the manufacturer’s instructions. The cDNA was stored at −20 °C for further analysis.

Five genes in the PI3K/AKT pathway were the focus of further analysis, including growth factor (*GF*), insulin receptor substrate 1 *(IRS1*), receptor tyrosine kinase (*RTK*), protein phosphatase 2A (*PP2A*) and *PTEN*, respectively. The sequence-specific primers were designed using the Primer Premier 5.0 software (PREMIER Biosoft International, Palo Alto, CA, USA). Primer sequences used in this study are provided in Supplemental Table S1. The relative expression levels of the five selected genes were determined by qRT-PCR in a Rotor-Gene Q Real-Time thermal cycler (Qiagen, Hilden, Germany) using the commercial kit SYBR^®^ Premix Ex TaqTM II (TaKaRa Bio Inc., Otsu, Shiga, Japan). The PCR procedure is implemented as follows: 95 °C for 3 min, followed by 40 cycles of 95 °C for 15 s and 55 °C for 30 s. A melting curve was obtained from 60 °C to 90 °C, with a 0.5 °C rise in temperature every 5 s to test the specificity of the amplified products. All results were standardized using actin genes, and relative expression was calculated using the 2^−ΔΔCt^ method [26].

### 2.4. Synthesis and Validation of dsRNA-Expressing Bacteria

The dsRNA was expressed in *Escherichia coli* HT115 with the expression vector L4440. Supplemental Table S1 lists the primers used in this study. The full-length fragment of *pten* was amplified from *L. dispar* cDNA.

The pGEM-T Easy (Promega, Madison, WI, USA) cloning vector was modified by sequential insertion of *PTEN* full-length fragments with two restriction endonucleases (SacI and HindIII). After sequencing the appropriate insertion of the cloning vector pGEM-*PTEN*, the L4440 and pGEM-*PTEN* plasmids were digested with the restriction endonucleases SacI and HindIII, subsequently resulting in the ligation of two fragments, *PTEN*-SacI/HindIII and L4440-SacI/HindIII. The plasmid L4440-*PTEN* was transformed into HT115 (DE3) using a standard CaCl_2_ transformation protocol [27]. The HT115 (DE3) containing plasmid L4440-*PTEN* was grown for 16 h at 37 °C in LB solid medium with ampicillin. PCR amplification of the bacterial solution and the double digestion technique were used to identify the insertion. Simultaneously, the empty vector L4440 was transformed into HT115 as a negative control. The isopropyl-β-D-thiogalactopyranoside (IPTG)-induced product was named dsRNA-L4440. The methodology for the synthesis of dsRNA-*PTEN* followed the protocol of the manufacturer. IPTG-induced L4440-*PTEN* contained HT115 cells that were pelleted by centrifugation. The cells were then resuspended in buffer (50 mM Tris and 10 mM EDTA, pH 7.5) [28]. This suspension was subjected to sonication (20 kHz for 15 min) and centrifuged at 9000 rpm for 20 min. The supernatant was stored at −20 °C until the next step.

### 2.5. Delivery of dsRNA via Feeding, Mortality and Behavioral Assays

Newly molting third-instar larvae were randomly divided into six groups: CK, dsRNA-L4440, dsRNA-*PTEN*, NPV, NPV + dsRNA-L4440 and NPV + dsRNA-*PTEN*. Each group included 100 larvae, and all tests were repeated three times. After the larvae molted, they were fed twice. The details of the treatments are listed in Table 1. The first feed was administered when they were newly molted, while the second was given 24 h later. After treatments, the larvae were reared in individual wells of a 24-well insect rearing box with ample artificial diet.

Mortality, time of death, the position prior to death and the length of the dead body were recorded. Multiple comparisons of treatments were calculated using the least squares means estimates. Student’s t test was performed for all pairwise comparisons. To clarify variations in the dead body after expression of the *PTEN* gene had been altered, and to understand the functions of *PTEN*, we further investigated the virion amount and the copies of the occlusion-derived virus (ODV) of LdMNPV in deceased larvae. The virion amount was processed by a hemacytometer, after extraction from larvae bodies, as described by Rohrmann [6]. The number of ODV copies was detected by qRT-PCR analysis, which was calculated by the *polyhedrin* gene (NCBI Reference Sequence: NC_001973.1). The primer sequences used in this study are provided in Supplemental Table S1.

## 3. Results

### 3.1. Preliminary Screening of Genes in PI3K/AKT Signaling Pathway

Based on our previous analysis of transcriptome data (Accession Number: SRX5290160-SRX5290168), the DEGs in the PI3K/AKT pathway and interrelated signaling pathways between groups were primarily focused on, analyzed separately and visualized by Cytoscape software (version 3.8.2), as shown in Supplemental Figure S1. As a stable intersection of signal transduction, the PI3K/AKT signaling pathway was activated by different types of growth factors and cellular stimuli regulated by upstream pathways, such as the insulin-like signaling pathway and chemokine signaling pathway. In addition, the PI3K/AKT signaling pathway was shown to regulate fundamental functions affecting transcription, cell cycle progression, growth and survival, by downstream pathways such as the mTOR signaling pathway, MAPK signaling pathway, FoxO signaling pathway, P53 signaling pathway and apoptosis [29,30,31]. Based on the gene expression characteristics in the PI3K/AKT signaling pathway, five genes (*GF, RTK, IRS1, PP2A* and *PTEN*) were the focus of further analysis.

### 3.2. Quantitative Real-Time PCR Analysis of Core Genes in the PI3K/AKT Pathway

**Figure 1:**
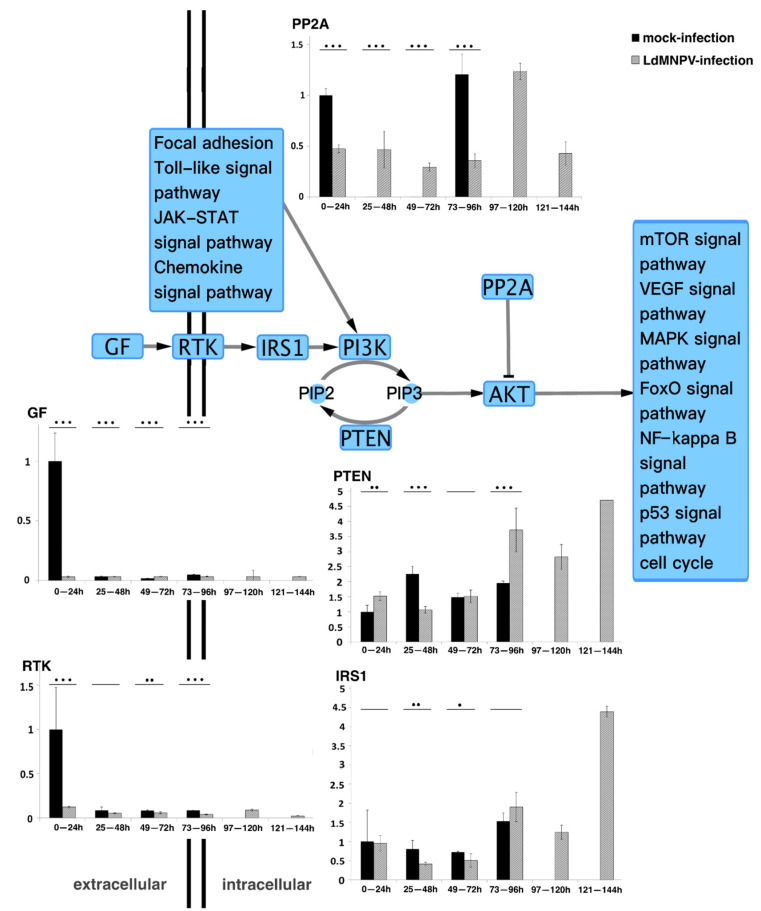
Quantitative real-time PCR analysis of core genes in PI3K/AKT pathway. GF, RTK and IRS1 are nutrient message receptors, which receive nutrient signals to regulate downstream phosphorylation signals [23]. PP2A is described as being responsible for the majority of serine/threonine phosphatase activity in eukaryotic cells and suppresses PI3K/AKT-generated signals by direct AKT dephosphorylation. This results in activation of additional transcription factors to express target genes [37]. PTEN, as a multifunction lipid phosphatase, is well known for dephosphorylating phosphatidylinositol-3, 4, 5-triphosphate (PIP3) to indirectly inhibit activation of AKT, which is involved in cell cycle regulation and cell differentiation [32,33,34,35,38,39]. * *p* < 0.05; ** *p* < 0.01; *** *p* < 0.001 for pairwise comparisons by Student’s *t*-test.

Five genes (*GF, RTK, IRS1, PP2A* and *PTEN*) in the PI3K/AKT pathway were further analyzed. The mRNA expression levels of the five genes in infected and uninfected *L. dispar* were assayed using qRT-PCR. As shown in Figure 1, the expression levels of *GF* and *RTK* were similarly low except for the mock-infected group which displayed high expression levels at the earlier stage. It is speculated that the high expression levels in the mock-infected group were stimulated by food intake, mirroring their normal behavior under natural conditions. The *PP2A* gene exhibited differences compared to the mock-infected group, and the expression pattern of the LdMNPV-infected group started at a low expression level and then sharply increased in the period of 97–120 hpi. The expression levels of *IRS1* and *PTEN* were similar. In the LdMNPV-infected group, the expression levels remained at a low level with a tiny undulation at the early stages and then drastically increased. Considering the high expression level only occurred in the later stage, the increase in *IRS1* gene expression did not affect the *PTEN* gene. In addition, *PTEN* also indirectly mediated intracellular signaling that was shown to regulate cellular processes and determined cell fate [32,33,34,35,36]. ODV production occurred later than 3 dpi. It is reasonable to assume that prolonging the infected larval instar duration facilitated an increase in the ODV output.

### 3.3. Synthesis and Validation of DsRNA-Expressing Bacteria

The full length of the *PTEN* gene was validated using 1% agarose gel (Supplemental Figure S1A). The insertion of the *PTEN* gene into the pGEM-T Easy cloning vector was validated using 1% agarose gel (Supplemental Figure S1B) and later verified by sequencing. L4440-*PTEN* was validated using 1% agarose gel (Supplemental Figure S1C).

### 3.4. Delivery of dsRNA via Feeding, Mortality Assays and Behavioral Assays

**Figure 2:**
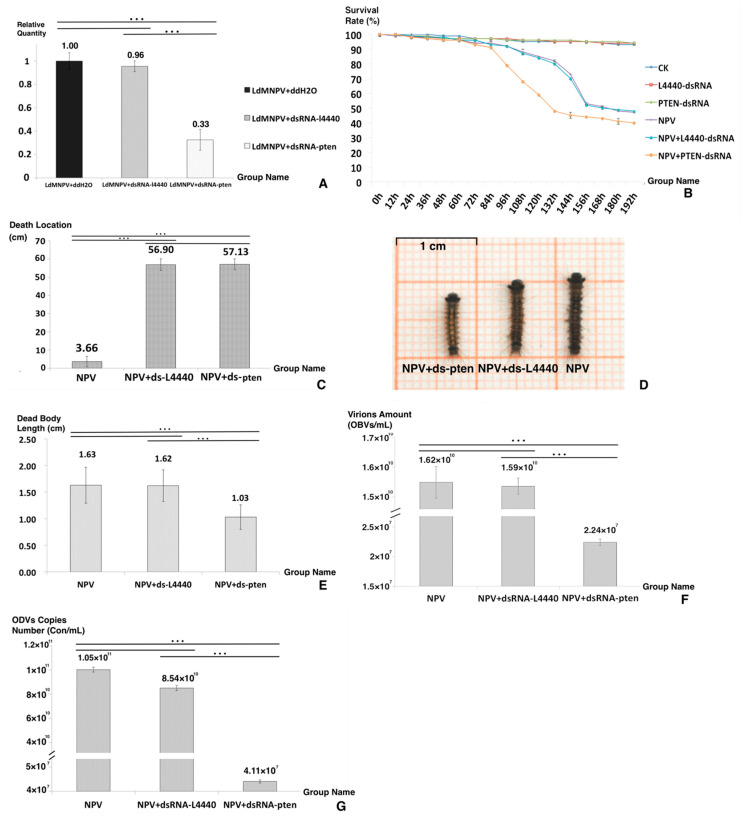
Bioassays after dsRNA feeding. (**A**) The knockdown rate of the *PTEN* gene. (**B**) The survival rate of the six groups. (**C**) The death location of the three groups. (**D**) The dead body appearance of each group. (**E**) The dead body length of the three groups. (**F**) The virion amount of the three groups. (**G**) The ODV copy number of the three groups. Asterisks indicate significant differences: *** *p* < 0.001 for pairwise comparisons by Student’s *t*-test.

The knockdown rate for the *PTEN* gene was 67.43% at 72 h after feeding dsRNA-*PTEN*, as shown in Figure 2A. All treated larvae belonging to the groups CK, dsRNA-L4440 and dsRNA-*PTEN* successfully molted into fourth-instar larvae. Compared to the CK group, the dsRNA-L4440 and dsRNA-*PTEN* groups did not exhibit significant differences in many aspects including behavior, food intake and growth rate. Afterwards, all larvae in the CK, dsRNA-L4440 and dsRNA-*PTEN* groups were raised to successfully emerge as adults and to mate and oviposit as normal. Compared to the NPV group, the NPV + dsRNA-L4440 group did not exhibit any significant differences in many aspects including mobility, time of death and appearance. However, the NPV + dsRNA-*PTEN* group displayed relatively large differences in many aspects.

Figure 2B,C display the differences between the NPV+ dsRNA-*PTEN* group and the other two control groups in survival rate and death location. When looking at the survival time (Figure 2B), that of the NPV+ dsRNA-*PTEN* group was shorter than that of the other two groups. It was concluded that the increase in *PTEN* expression was necessary to host death postponement. For death location (Figure 2C), the NPV+ dsRNA-*PTEN* group had a value of 3.66 (±3.01) cm, and the other two groups’ values were 57.13 (±2.89) cm for NPV and 56.90 (±3.16) cm for NPV + dsRNA-L4440. It was concluded that the NPV-induced climbing behavior virtually disappeared after knockdown of the *PTEN* gene.

Regarding the death appearance of the three groups (Figure 2D), the NPV and NPV + dsRNA-L4440 groups displayed typical death symptoms, such as liquidation, melanism and fragility, while the NPV + dsRNA-*PTEN* group displayed a bright color and a constrictive dead body that was significantly different from the two other groups. The disintegration of larval tissues caused by baculoviruses was the primary reason for the increase in dead body length [6,32]. The lengths of the dead bodies from the three groups are shown in Figure 2E: the NPV + dsRNA-*PTEN* group had a length of 1.03 (±0.23) cm, and the other two groups had lengths of 1.63 (±0.33) cm for NPV and 1.62 (±0.29) cm for NPV + dsRNA-L4440. In the NPV + dsRNA-*PTEN* group, liquefaction did not occur in the corpses. Moreover, the larvae of the NPV + dsRNA-*PTEN* group displayed a deferred response to environmental stimuli and bradykinesia. Based on these results and observation of larval behavior, it was hypothesized that ODV production was interrupted in the NPV + dsRNA-*PTEN* group.

To detect the effect of LdMNPV replication, we counted the virion amount and ODV copy numbers (Figure 2F,G). As shown in Figure 2F, the virion amount for the NPV + dsRNA-*PTEN* group was 2.240 *×* 10^7^ OBs/mL, and for the other two groups, it was 1.624 *×* 10^10^ OBs/mL for NPV and 1.592 *×* 10^10^ OBs/mL for NPV + dsRNA-L4440. As shown in Figure 2G, the ODV copy number of the NPV + dsRNA-*PTEN* group was 4.11 *×* 10^7^ Con/mL, and for the other two groups, it was 1.05 *×* 10^11^ Con/mL for NPV and 8.54 *×* 10^10^ Con/mL for NPV + dsRNA-L4440. The results of the virion amount and ODV copy numbers verify that the NPV + dsRNA-pten group was different in comparison to the other two groups, and at the late period of infection, the increased expression of the *PTEN* gene was important to maintain ODV maturation. It is reasonable to assume that the increase in *PTEN* gene expression at a later stage was necessary for prolonging the infected larval instar duration, ODV production and NPV-led climbing behavior.

## 4. Discussion

Baculoviruses have been shown to induce a series of behavioral changes in infected larvae including prolonged instars and the behavior of climbing to an elevated location before death [4,5,6]. In the last decade, some of the primary genes responsible for NPV manipulation have been identified, providing insight into NPV-led influences on the host even when depending on just one gene [6,11,12,13,14,15,16,17,19,21,40,41]. However, the details of the interactions between the host and baculovirus have rarely been explored. In this study, based on the results of qRT-PCR of essential genes in the PI3K/AKT pathway, we discovered that at the terminus of infection, the expression of the *PTEN* gene was continuously elevated, and therefore the *PTEN* gene was selected for knockdown. The results of RNA interference revealed that the high expression level of the *PTEN* gene was required for a prolonged host duration, high virion production and the climbing behavior induced by LdMNPV. This is the first direct evidence indicating that the PI3K/AKT pathway and *PTEN* gene are involved in LdMNPV production and host manipulation.

In rapidly replicating viruses, the cellular hyperactivation is sufficient to increase viral titers [42,43]. The NPVs are also capable of altering host cellular metabolism in a variety of ways to increase transmission rates. Moreover, since Munger et al. reported the first eukaryotic virus tampering with cellular metabolism, more viruses have been recognized as able to reprogram cellular processes by hijacking host cell signals, especially phospholipids, so as to manipulate host cellular nutrient utilization and guarantee the required micro-environment for replication [42,44,45,46,47,48]. The phospholipid signal has been shown to be involved in endocytic and exocytic processes, accepting transporter signals, coordinating vesicular trafficking and modulating metabolism [49,50].

Insects are able to receive and endure environmental stress and work through cellular signals to regulate physiological processes, such as postembryonic development, reproduction and ecdysis. During their lifecycle, with corresponding stimulation, the CNS secretes hormones such as steroids, 20E and JH, to activate the insulin-like signaling pathway and downstream phosphorylation signals, such as AKT, FoxO and TOR, to trigger an appropriate response [22]. Moreover, the insulin-like/PI3K/AKT signal has been shown to play an important role in insect diapause, energy storage, cellular regulation and lifespan [23]. In *Leptinotarsa decemlineata*, It was documented that the knockdown of both *PI3K* and *IRS1* decreased the expression of JH signaling genes, such as *Hairy*, Juvenile Hormone Acid Methyltransferase (*JHAMT*) and Kruppel Homolog 1 (*Kr-h1*), and 20E signaling genes, such as Ecdysone Receptor (*EcR*), Ecdysone-induced Protein 75 (*E75*) and Hormone Receptor 3 (*HR3*), resulting in altered larval development and growth inhibition [23].

A number of pathogens, particularly viruses, have been recognized to reprogram cellular processes by hijacking host cellular signaling to create the required environment for their propagation. The signal transduction pathways are the connection between larvae receiving environmental stimuli and the corresponding cellular response. As the core hinge of the messaging cascade, the PI3K/AKT pathway administers metabolic responses and growth promotion, including transcription factors and other kinase signaling proteins that affect cell cycle entry, cell growth and survival, hence the balance between dephosporylation and phosphorylation of several critical substrates [51]. Relying on the gene expression characteristics of the PI3K/AKT signaling pathway, five genes (*GF, RTK, IRS1, PP2A* and *PTEN*) were the focus of further gene expression analysis by qRT-PCR. Each of these genes displayed a unique expression pattern that was dependent on the cell fate. Strikingly, the qRT-PCR analysis showed that in the LdMNPV-infected group, the *PTEN* gene was expressed at a low level with a tiny undulation until 3 dpi, then drastically increasing from 4 dpi. The PTEN protein indirectly mediated intracellular signaling, regulated cellular processes and determined cell fate [32,33,34,35,36]. Moreover, PIP3, a second messenger affecting cell fate, is dependent on the catalysis of PTEN and PI3K to regulate downstream signaling [39]. Reports had shown that the persistent high-level expression of the *PTEN* gene caused pausing of the cell cycle [32,33,34,35,36]. In addition, it has been suggested that the *ptp* gene is required for inducing the climbing behaviors in group I alphabaculoviruses, including BmNPV and AcMNPV [8,12]. As a group II alphabaculovirus, LdMNPV has a larger genome than many others, but it lacks the *ptp* gene [52]. The PTEN protein is a multifunctional lipid phosphatase belonging to the PTP family. In addition, recent research suggested that PTEN is involved in the effect of myo-inositol (MI) on aging and lifespan [53], which is a typical behavioral change of infected *L. dispar* larvae. These results suggest that when compared to other genes, the expression pattern of *PTEN* increases significantly in the latter period and may play a more important role. The above results imply that the persistent high expression of the *PTEN* gene during the terminus of infection might be involved in infected host behavioral change and viral replication.

In summary, we monitored the expression patterns of five candidate genes (*GF, RTK, IRS1, PP2A* and *PTEN*) during the infection process and explored the role of the PI3K/AKT pathway. Subsequently, the *PTEN* gene was knocked down, and the NPV + dsRNA-*PTEN* treated group showed significant differences in death location, survival rate, dead body characteristics and ODV production in the NPV and NPV + dsRNA-L4440 groups. We have demonstrated that the fluctuation in the PI3K/AKT signal pathway and increased expression of the *PTEN* gene are necessary for virion production, host death postponement and the climbing behavior of terminally infected larvae. Further research into other relevant pathways is required to fully reveal the complicated mechanism behind this behavioral change. Overall, these results suggest that the PI3K/AKT pathway and *PTEN* gene might play an essential role in the LdMNPV-activated “tree-top disease”.

## Figures and Tables

**Table 1:** Treatments of delivery dsRNA via feeding.

Group Name	First Feed	Second Feed
CK	Isopyknic dd-H_2_O	Buffer (dsRNA) ^1^
dsRNA-L4440	Isopyknic dd-H_2_O	dsRNA-L4440
dsRNA-*PTEN*	Isopyknic dd-H_2_O	dsRNA-*PTEN*
NPV	10^6^ OBs/larva of LdMNPV	Buffer (dsRNA)
NPV + dsRNA-L4440	10^6^ OBs/larva of LdMNPV	dsRNA-L4440
NPV+ dsRNA-*PTEN*	10^6^ OBs/larva of LdMNPV	dsRNA-*PTEN*

^1^ Buffer (dsRNA) contained 50 mM Tris and 10 mM EDTA, pH 7.5.

## Data Availability

The transcriptome data (Accession Number: SRX5290160-SRX5290168) are available in the Genbank database.

## Supplementary Materials

The following supporting information can be downloaded at: www.mdpi.com/article/10.3390/genes13020247/s1, Figure S1: The DEGs in PI3K/Akt and interrelating signal pathways after LdMNPV infection; Figure S2: Analysis of agarose gel electrophoresis; Table S1: The sequence of qPCR primers.
