# Supplementary material for: The PI3K/AKT Pathway and PTEN Gene Are Involved in “Tree-Top Disease” of Lymantria dispar
Source: Genes (Basel). 2022 Jan 27;13(2):247. doi: 10.3390/genes13020247 (PMC8871656; doi:10.3390/genes13020247)
Supplement: Supplementary file 1 [file genes-13-00247-s001.zip › genes-1543644-supplementary.pdf]

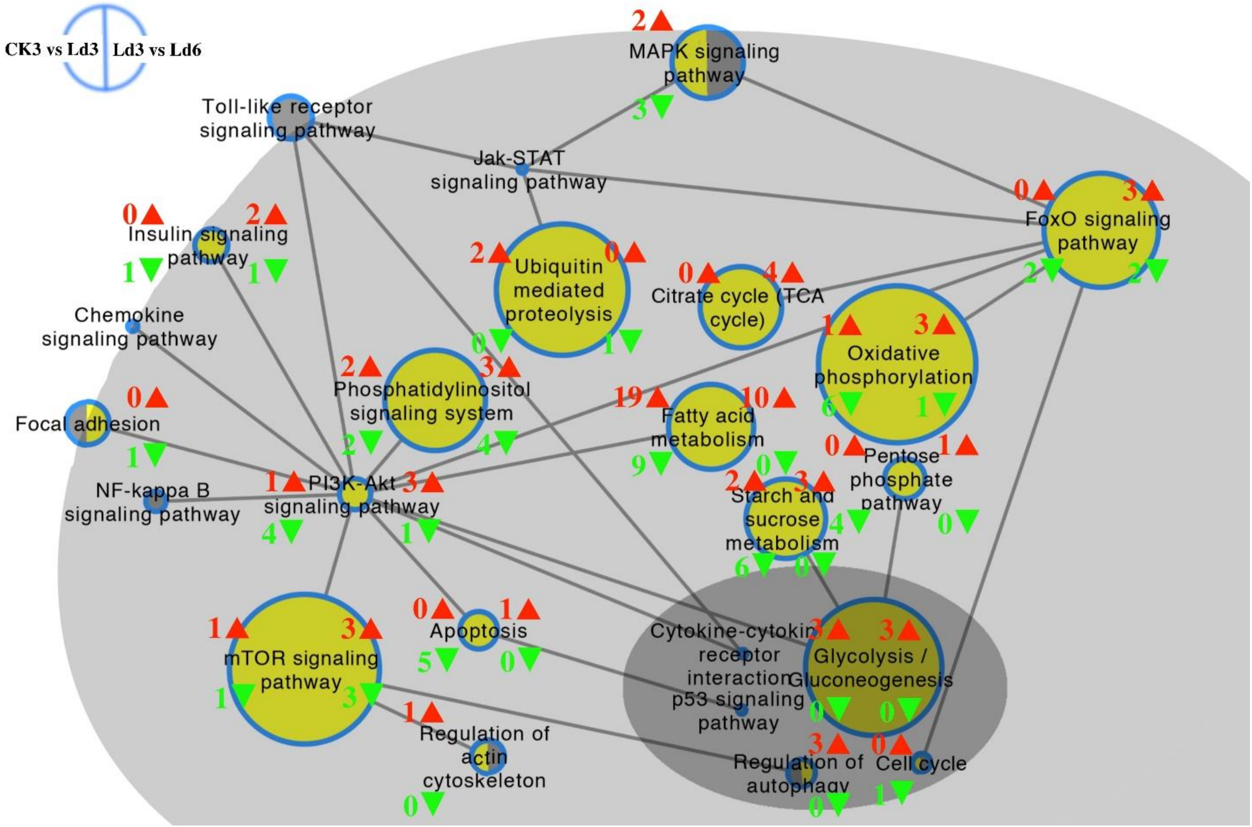


**Figure S1.** The DEGs in PI3K/Akt and Interrelating Signal Pathways after LdMNPV infection. The changed pathways were painted yellow, and the amount of changing genes listed out beside. Left half of circle stands for comparison between 3 days post infection larvae and 3 days post mock-infection larvae, and right half of circle stands for comparison between 3 days post and 6 days post infection larvae.

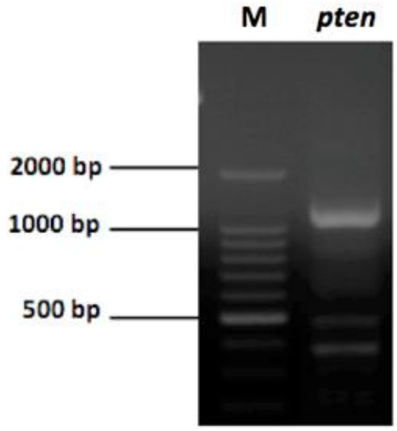


(**a**)

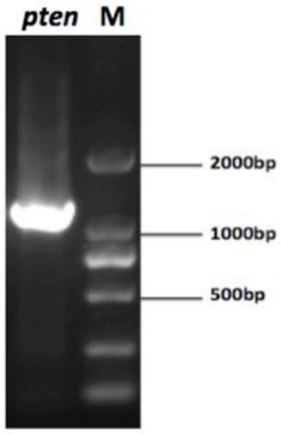


(**b**)

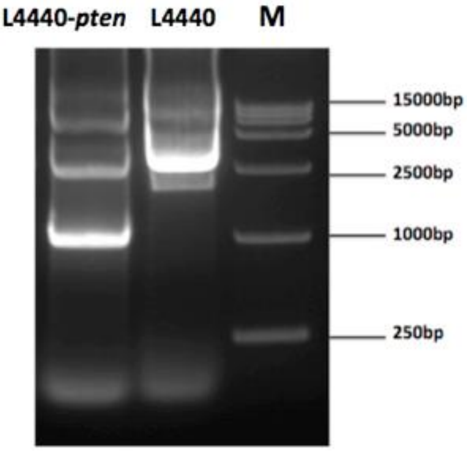


(c)

**Figure S2.** Analysis of agarose gel electrophoresis. **(A)** 1% Agarose Gel Electrophoresis of the full length of *pten* genes (M strands for Marker, *pten* stands for pten gene). **(B)** 1% Agarose Gel Electrophoresis of pGEM-T-*pten* plasmid (M strands for Marker, *pten* stands for *pten* gene). **(C)** Double Digestion of *pten* Gene insert into L4440 plasmid (M strands for Marker, L4440-*pten* strands for *pten* gene insert into L4440 plasmid, L4440 strands for empty L4440 plasmid).

**Table S1.** Treatments of delivery dsRNA via feeding.

| **Primer name** | **Sequences（5′-3′）** |
|---|---|
| GF-QPCR-F | AGGGGAAAAGCGAGGCGGAC |
| GF-QPCR-R | AGTTCCTGTGGCGTGGTGGG |
| RTK-QPCR-F | GTGCGATGGATGAGCCCCG |
| RTK-QPCR-R | CGACCACGTAGCGCACCAC |
| IRSI-QPCR-F | CAAGGGGCTATGCGTGGGC |
| IRSI-QPCR-R | GCTGCCTCGACACCTTCGG |
| PTEN-QPCR-F | CAGCGGGCAGAAAGCGACC |
| PTEN-QPCR-R | GACACGGTAAGCTCCGGCG |
| PP2A-QPCR-F | CTGCGTGCGTTGCGTACAGG |
| PP2A-QPCR-R | GTGCTCGCGCCTCGAACTTG |
| *pten*-F | ATGGGCTTCCCAGCGGAGAG |
| *pten*-R | TCACAAGTACGTTGATTCACTTGA |
| *pten-Sac Ⅰ*-F | GAGCTCATGGGCTTCCCAGCG |
| *pten-Hind*Ⅲ-*R* | TTCGAATCACAAGTACGTTGATTC |
| LdMNPV-polyhedrin-QPCR-F | CAACTGGAGCGGCAAAGAGT |
| LdMNPV-polyhedrin-QPCR-R | ACTCGTTGTTCTCGACCGTG |
